# Engraftment outcome of patients with anti-HLA antibodies in HLA-mismatched peripheral blood stem cell transplantation

**DOI:** 10.1007/s12185-025-03952-y

**Published:** 2025-03-20

**Authors:** Takeshi Hagino, Kazuhiro Ikegame, Hidenori Tanaka, Yoshinobu Kanda, Katsuji Kaida, Takahiro Fukuda, Yukio Kondo, Maho Sato, Noriko Doki, Hirohisa Nakamae, Ken-ichi Matsuoka, Yasuo Mori, Hideki Sano, Tetsuya Eto, Toshiro Kawakita, Yoshiko Hashii, Tatsuo Ichinohe, Yoshiko Atsuta, Junya Kanda, the HLA Working Group of Japanese Society for Transplantation, Cellular Therapy

**Affiliations:** 1 Department of Hematology, Tama-Hokubu Medical Center, Tokyo Metropolitan Health and Medical Treatment Corporation, Tokyo, Japan; 2 Hematopoietic Cell Transplantation Center, Aichi Medical University of School of Medicine, 1-1 Yazakokarimata, Nagakute, Aichi 480-1195 Japan; 3 HLA Foundation Laboratory, Kyoto, Japan; 4 Division of Hematology, Saitama Medical Center, Jichi Medical University, Saitama, Japan; 5 Department of Hematology, Hyogo Medical University Hospital, Hyogo, Japan; 6 Department of Hematopoietic Stem Cell Transplantation, National Cancer Center Hospital, Tokyo, Japan; 7 Department of Hematology, Toyama Prefectural Central Hospital, Toyama, Japan; 8 Department of Hematology/Oncology, Osaka Women’s and Children’s Hospital, Osaka, Japan; 9 Hematology Division, Tokyo Metropolitan Cancer and Infectious Diseases Center, Komagome Hospital, Tokyo, Japan; 10 Department of Hematology, Osaka Metropolitan University Hospital, Osaka, Japan; 11 Department of Hematology and Oncology, Okayama University Hospital, Okayama, Japan; 12 Hematology, Oncology & Cardiovascular Medicine, Kyushu University Hospital, Fukuoka, Japan; 13 Department of Pediatric Oncology, Fukushima Medical University Hospital, Fukushima, Japan; 14 Department of Hematology, Hamanomachi Hospital, Fukuoka, Japan; 15 Department of Hematology, National Hospital Organization Kumamoto Medical Center, Kumamoto, Japan; 16 Department of Pediatrics, Osaka International Cancer Institute, Osaka, Japan; 17 Department of Hematology and Oncology, Research Institute for Radiation Biology and Medicine, Hiroshima University, Hiroshima, Japan; 18 Japanese Data Center for Hematopoietic Cell Transplantation, Aichi, Japan; 19 Department of Registry Science for Transplant and Cellular Therapy, Aichi Medical University School of Medicine, Aichi, Japan; 20 Department of Hematology and Oncology, Graduate School of Medicine, Kyoto University, Kyoto, Japan; 21 Present Address: Department of Hematology, Endocrinology and Metabolism, Institute of Biomedical Sciences, Tokushima University Graduate School, Tokushima, Japan

**Keywords:** Anti-HLA antibody, DSA, PBSCT, Engraftment, MFI

## Abstract

Anti-human leukocyte antigen (HLA) antibodies, particularly donor-specific HLA antibodies (DSA), negatively impact engraftment in hematopoietic cell transplantation. Past studies have proposed various interventions to reduce DSA, but these were primarily from single centers and not from large-scale registry data. Therefore, we conducted a retrospective analysis of nationwide registry data to examine the effects of anti-HLA antibodies on engraftment. Evaluable patients were classified into an anti-HLA antibody-negative group (*n* = 3657), an anti-HLA antibody-positive group (without high DSA) (*n* = 137), and a high-DSA (MFI > 5000) group (*n* = 8). Patient characteristics differed significantly between the anti-HLA antibody-negative and anti-HLA antibody-positive groups, and the number of patients with DSA was lower than expected. Statistical analyses revealed that the anti-HLA antibody-positive group had better neutrophil engraftment than the anti-HLA antibody-negative group (94.0% vs 84.2%, *p* < 0.001) but worse platelet engraftment (60.3% vs 64.9%, *p* = 0.047). In the high DSA group, two patients received a DSA-depleting intervention. Only one patient with an MFI of 5832 (without intervention) developed primary graft failure, while the remaining seven achieved engraftment. In this study, the effect of anti-HLA antibodies remained inconclusive, and the possibility of neutrophil engraftment with high-DSA levels was confirmed.

**Supplementary Information:**

The online version contains supplementary material available at 10.1007/s12185-025-03952-y.

## Introduction

As human leukocyte antigens (HLA)-mismatched hematopoietic stem cell transplantation has become more widespread, anti-HLA antibodies in pre-transplant recipients have come into focus. We and others have reported that anti-HLA antibodies, especially donor-specific anti-HLA antibodies (DSA), are a risk factor for graft failure not only in cord blood transplantation [1] but also in HLA-mismatched (haploidentical) stem cell transplantation [2, 3, 4, 5]. Various protocols have been proposed to reduce DSA levels, such as suppression of antibody production using rituximab [6], depletion of DSA through transfusion of targeted HLA-expressing platelets [7], and immunomodulation using high-dose intravenous immunoglobulin therapy [8]. The consensus guidelines from the European Society for Blood and Marrow Transplantation (EBMT) propose that a mean fluorescence intensity (MFI) of 5,000 or higher is the cut-off for high DSA [9]. However, the proposition that anti-HLA antibodies are a risk factor for graft failure has been reported primarily in single centers and has not yet been reported in large registry data. Additionally, none of the DSA-depleting interventions have sufficient evidence and are not covered by medical insurance in Japan. Therefore, we identified patients with anti-HLA antibodies, particularly those with high levels of DSA, in nationwide registry data to elucidate the effect of anti-HLA antibodies on engraftment in practical settings. To exclude the influence of the stem cell source, we focused on peripheral blood stem cell transplantation (PBSCT) in this study.

The effect of anti-HLA antibodies on engraftment is inconclusive based on the current data. However, it might be beneficial to know the existence of some patients who achieved neutrophil engraftment even with high levels of DSA. We also discussed why the MFI value of DSA is sometimes consistent with expectations and sometimes not, based on recent literature.

## Materials and methods

### Patients

**Fig. 1:**
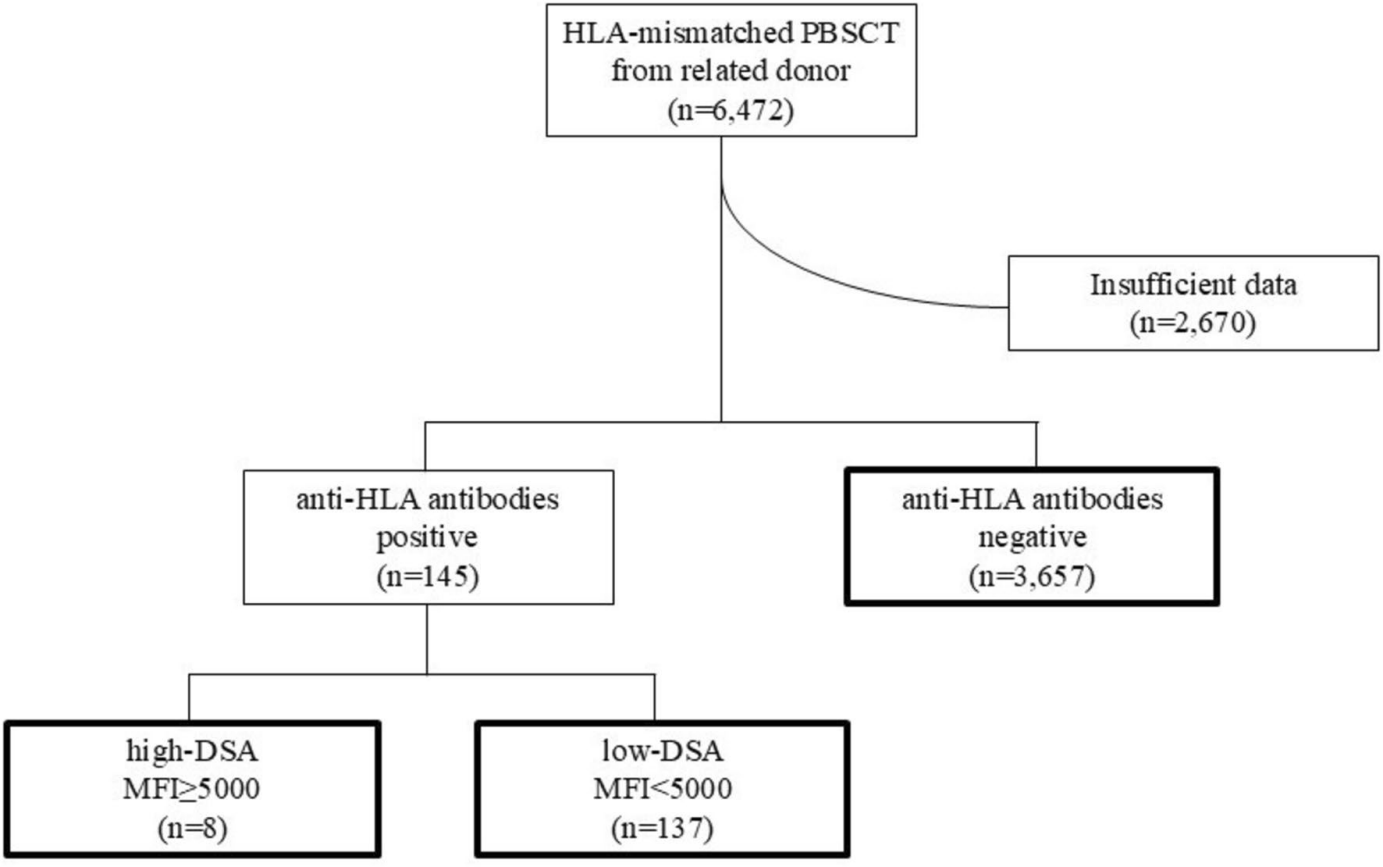
Consort diagram. Consort diagram for grouping of “anti-HLA antibodies negative,” “high DSA,” and “anti-HLA antibody-positive (low DSA)” is shown. HLA, human leukocyte antigen; DSA, donor-specific antibody

A total of 6,472 patients who had undergone HLA-mismatched PBSCT from a related donor were enrolled in the Japanese Society for Transplantation and Cellular Therapy/The Japanese Data Center for Hematopoietic Cell Transplantation (JSTCT/JDCHCT) Transplant Registry Unified Management Program (TRUMP) during the 5 years from 2010 to 2014. Registration in the TRUMP requires checking one of three boxes: anti-HLA antibody-positive, anti-HLA antibody-negative, and not tested; if positive, checking whether it is DSA. We conducted a secondary survey for each DSA-positive case to determine the MFI of the DSA. According to the EBMT consensus, [9] we defined the cut-off for high DSA as MFI > 5,000. After excluding cases with insufficient data, 145 patients were found to have anti-HLA antibodies before transplantation, and the remaining 3,657 patients were classified into the anti-HLA antibody-negative group. Among the 145 patients with anti-HLA antibodies, only eight patients had high DSA (MFI > 5,000; high-DSA group). We designated the remaining 137 patients as anti-HLA antibody-positive (low DSA of less than MFI 5,000; not a high DSA of MFI > 5,000). No cases with DSA for multiple HLA loci were found. A consort diagram is shown in Fig. 1. We then compared the three groups: the high-DSA group (*n* = 8), the anti-HLA antibody-positive group (*n* = 137), and the anti-HLA antibody-negative group (*n* = 3,657). In this analysis, we evaluated antibodies targeting the HLA-A, B, C, and DRB1 loci based on the available data. Anti-HLA antibodies against HLA-DP, HLA-DQ, and HLA-DRB3/4/5 were not considered anti-HLA antibodies, because the donor data for these HLA loci were unavailable. MFI for each HLA allele was determined using a flow bead-based Luminex assay by the HLA Foundation Laboratory (*n* = 77), the Japanese Red Cross Society (*n* = 12), Repro CELL Incorporated (*n* = 5), cord blood bank (*n* = 1), the treating institution (n = 48), and unknown (*n* = 2).

## Statistics

Neutrophil and platelet engraftments were defined as an absolute neutrophil count of > 0.5 × 10^9^/L on the first of 3 consecutive days and a platelet count of > 20 × 10^9^/L without transfusion. The cumulative incidences of neutrophil and platelet engraftment were estimated by treating death before engraftment as a competing risk and were compared using the Gray test. Yates’ Chi-square test and Student’s t test were used to compare categorical and numerical variables (age and infused CD34^+^ cells), respectively, between the two groups. Given that comparing among all three groups was deemed to lack logical significance, the comparisons were restricted to pairs of groups with a predetermined statistical significance level of *p* < 0.05, owing to the exploratory nature of the study. Statistical analyses were performed using EZR (Saitama Medical Centre, Jichi Medical University; http://www.jichi.ac.jp/saitama-sct/SaitamaHP.files/statmedEN.html), a graphical user interface for R (version 3.0.2; R Foundation for Statistical Computing, Vienna, Austria) [10]. This study was approved by the Institutional Review Boards of the JSTCT and Tama-Hokubu Medical Center, Tokyo Metropolitan Health and Medical Treatment Corporation.

## Results

### Patients’ characteristics

The characteristics of the patients in the anti-HLA antibody-negative, anti-HLA antibody-positive, and high-DSA groups are shown in Table 1. Compared with the anti-HLA antibody-negative group, the anti-HLA antibody-positive group was predominantly female, older in age, and had different diseases. Additionally, the anti-HLA antibody-positive group had a predominance of remission state, high Hematopoietic Cell Transplantation-specific Comorbidity Index (HCT-CI) scores, second or further transplantation, ABO mismatch, and HLA mismatches in the Graft versus Host (GVH) and Host versus Graft (HVG) directions, but fewer infused CD34^+^ cells. Furthermore, reduced-intensity conditioning without total body irradiation followed by GVH disease (GVHD) prophylaxis not consisting of mycophenolate mofetil (MMF), methotrexate (MTX), or post-transplant cyclophosphamide (PTCy) but consisting of tacrolimus (TAC) plus corticosteroid was more used in the anti-HLA antibody-positive group than in the anti-HLA antibody-negative group. Anti-thymocyte globulin was used as GVHD prophylaxis 0, 2, and 80 in the high-DSA, 2 anti-HLA antibody-positive (low-DSA), and 80 anti-HLA antibody-negative groups, respectively. We discuss the differences in the background and their effects on the following results.

**Table 1:** Patients’ characteristics

Covariates		Anti-HLA Ab negative (*n* = 3,657)	Anti-HLA Ab positive (*n* = 137)	High-DSA (MFI > 5,000) (*n* = 8)	*p* value (neg. vs pos.)	*p* value (neg. vs DSA)	*p* value (pos. vs DSA)
Sex	Male	2,143	58	1	< 0.001	0.022	0.193
	Female	1,514	79	7			
Age (years)	Average	37.1	46.9	37.9	< 0.001	0.912	0.092
	(range)	(0 – 76)	(3 – 68)	(18 – 47)			
PS	0–1	2,452	98	7	0.531	0.688	0.653
	2–4	848	39	1			
Disease	MDS/AML	1687	98	7	< 0.001	0.326	0.923
	ALL	722	19	1			
	Other leukemia	189	5	0			
	CML/MPD	145	3	0			
	Lymphoma	602	9	0			
	Miscellaneous	285	2	0			
Disease status	Remission	440	102	7	< 0.001	0.002	0.783
	Non-remission	942	31	1			
HCT-CI	0–2	3,166	86	7	< 0.001	1.000	0.186
	3–10	422	64	1			
History of previous stem cell transplantation	0	2,221	79	6	0.384	0.692	0.638
	1	1,147	47	2			
	2	236	11	0			
	3–6	52	0	0			
Conditioning regimen	MAC	1,113	32	3	< 0.001	1.000	0.628
	RIC	1622	105	5			
	TBI (-)	1,784	92	7	< 0.001	0.071	0.417
	TBI ( +)	1,831	45	1			
Sex combination (Pt ← Donor)	match	1,830	78	1	0.092	0.002	0.011
	M ← F	971	26	1			
	F ← M	748	33	6			
ABO type combination	Match	1,686	78	5	< 0.001	1.000	0.677
	Major-mismatch	621	33	2			
	Minor-mismatch	57	20	0			
	Major + minor mismatch	201	8	1			
GVHD prophylaxis	Cyclosporin-based	1,142	15	0	< 0.001	0.115	0.695
	Tacrolimus-based	2,394	122	8			
Additional GVHD prophylaxis	Corticosteroid (-)	2,493	75	0	< 0.001	< 0.001	0.008
	Corticosteroid ( +)	1,057	62	8			
	MMF (-)	2,980	109	7	< 0.001	1.000	0.927
	MMF ( +)	338	28	1			
	MTX (-)	1,500	100	8	< 0.001	0.003	0.198
	MTX ( +)	2,066	37	0			
	post-CY (-)	3,506	125	8	0.005	1.000	0.83
	post-CY ( +)	136	12	0			
HLA mismatch in GVH vector	0	475	9	0	< 0.001	0.107	0.741
	1–2	2,020	58	3			
	3–	1,078	70	5			
HLA mismatch in HVG vector	0	372	3	0	< 0.001	0.076	0.405
	1–2	2,003	62	2			
	3–	1,197	72	6			
Infused CD34^+^ cells (× 10^6^/kg)	Average	5.33	3.97	4.31	< 0.001	0.515	0.739
	(range)	(0.01–52.30)	(0.74–15.10)	(1.31–10.49)			

HLA, human leukocyte antigen; DSA, donor-specific anti-HLA antibody; MFI, median fluorescence intensity; HCT-CI, Hematopoietic Cell Transplantation-specific Comorbidity Index; PS, performance status; Pt, patient; GVH, in graft-versus-host direction; HVG, in host-versus-graft direction; GVHD, graft-versus-host disease; MDS, Myelodysplastic syndrome; AML, Acute myelogenous leukemia; ALL, Acute lymphoblastic leukemia; CML, Chronic myeloid leukemia; MPD, Myeloproliferative Disease; RIC, Reduced-intensity conditioning; MAC, myeloablative conditioning; TBI, Total body irradiation; M, male; F, female; MMF, mycophenolate mofetil; MTX, methotrexate; Post-CY, post-transplant cyclophosphamide

Characteristics of patients in the anti-HLA antibody-negative, anti-HLA antibody-positive, and high-DSA groups are shown in the three left columns. Background covariates between the anti-HLA antibody-negative and anti-HLA antibody-positive groups, anti-HLA antibody-negative and high-DSA groups, and the anti-HLA antibody-positive and high-DSA groups were compared. The p values of Yates’ Chi-squared test for categorical variables or Student’s t test for age and infused CD34 + cells are shown in the three right columns. Significant differences (*p* < 0.05) are highlighted in red

### Neutrophil and platelet engraftments

**Fig. 2:**
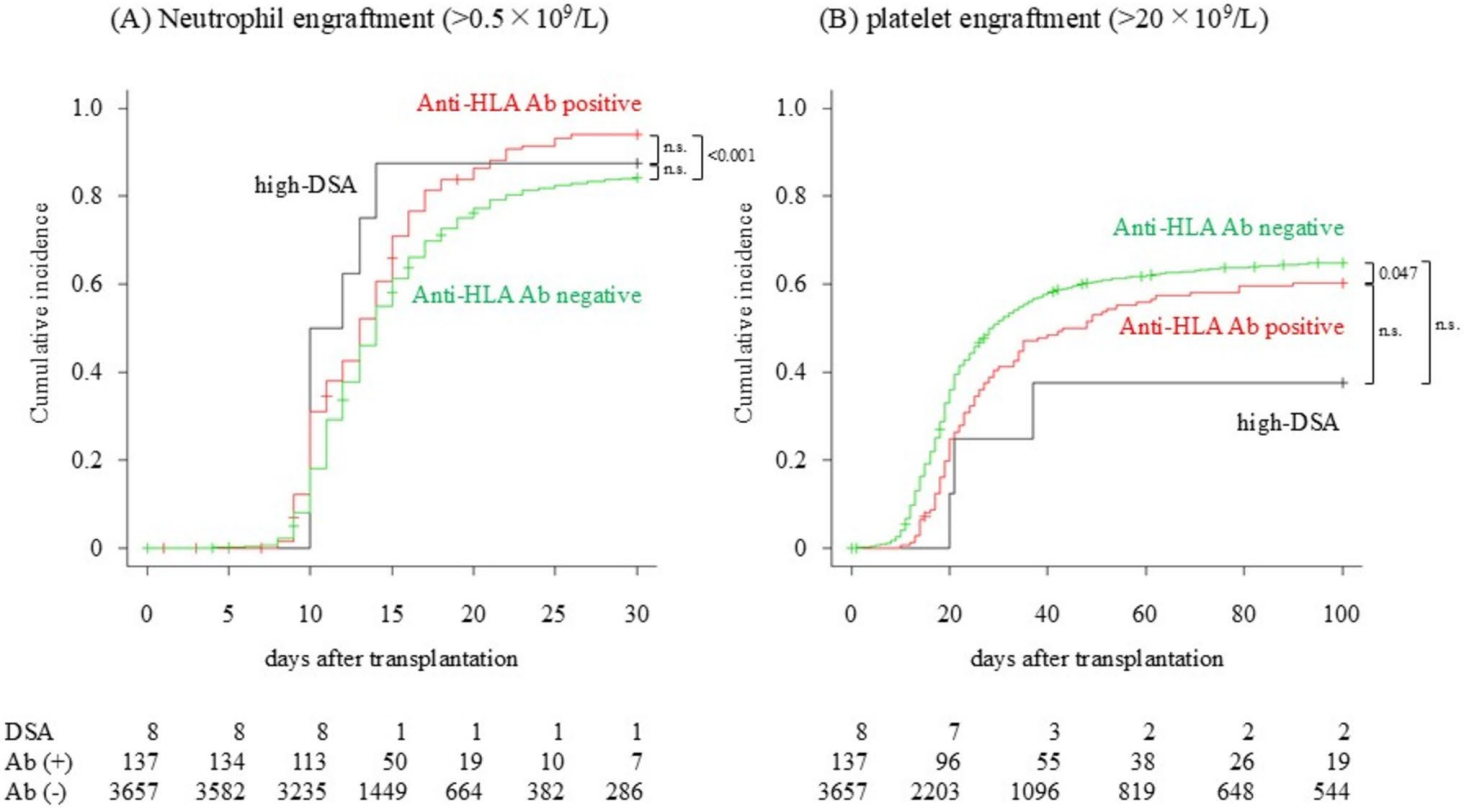
Neutrophil and platelet engraftments. Cumulative incidences of neutrophil (**A**) and platelet (**B**) engraftment are described in three groups: anti-HLA antibody-negative group (*n* = 3,657, green), anti-HLA antibody-positive group (*n* = 137, red), and high-DSA group (*n* = 8, black). Death before engraftment was defined as the competing risk. The at-risk numbers are shown in the figure. HLA: human leukocyte antigen; DSA, donor specific anti-HLA antibody; DSA, high-DSA group; Ab ( +), anti-HLA antibody-positive group; Ab (-), anti-HLA antibody-negative group

The cumulative incidence of neutrophil and platelet engraftment is shown in Fig. 2. The estimates of neutrophil and platelet engraftment rates for the anti-HLA antibody-negative, anti-HLA antibody-positive, and high-DSA groups and the statistical differences are summarized in Table 2. Contrary to previous reports, our data revealed the following: the cumulative incidence of neutrophils on day 30 was higher in the anti-HLA antibody-positive group than in the anti-HLA antibody-negative group (94.0% vs. 84.2%, *p* < 0.001); the cumulative incidence of platelets on day 100 was higher in the anti-HLA antibody-negative group than in the anti-HLA antibody-positive group (64.9% vs. 60.3%, *p* = 0.047); and high levels of DSA (MFI > 5,000) did not significantly affect the neutrophil and platelet engraftment outcome compared with the other two groups according to monovariate analyses. The first two results are conflicting and are difficult to interpret or explain; they will be discussed later. The last result is probably because the number of patients in the high-DSA group was too small to provide sufficient statistical power. Therefore, we focused on clarifying the actual outcomes in the DSA-high group.

**Table 2:** Estimation of neutrophil and platelet engraftments

Engraftment rate	estimates	Anti-HLA Ab negative (*n* = 3,657)	Anti-HLA Ab positive (*n* = 137)	High-DSA (MFI > 5,000) (*n* = 8)	*p* value (neg. vs pos.)	*p* value (neg. vs DSA)	*p* value (pos. vs DSA)
Neutrophil	Point estimate	0.842	0.940	0.875	< 0.001^a^	0.111	0.656
	95%CI	0.830–0.853	0.877–0.912	0.174–0.990			
Platelet	Point estimate	0.649	0.603	0.375	0.047^b^	0.132	0.267
	95%CI	0.633–0.664	0.515–0.680	0.070–0.697			

HLA, human leukocyte antigen; DSA, donor specific anti-HLA antibody; SCT, stem cell transplantation; MFI, median fluorescence intensity; GVH, in graft-versus-host direction; HVG, in host-versus-graft direction; NC, Nucleated cells; Plt, platelets; AML, Acute myelogenous leukemia; ALL, Acute lymphoblastic leukemia; non-CR, non-complete remission; F, female; M, male

Point estimates with 95% confidence intervals of neutrophil and platelet engraftment rates in the anti-HLA antibody-negative, anti-HLA antibody-positive, and high-DSA groups are shown in the three left-hand columns. Statistical differences in the Gray test between the anti-HLA antibody-negative and anti-HLA antibody-positive groups, anti-HLA antibody-negative and high-DSA groups, and anti-HLA antibody-positive and high-DSA groups are shown in the three right columns. Significant differences (*p* < 0.05) are highlighted in red

^a^The engraftment rate of neutrophils was higher in the anti-HLA antibody-positive group than in the anti-HLA antibody-negative group

^b^The engraftment rate of platelets was higher in the anti-HLA antibody-negative group than in the anti-HLA antibody-positive group

### Profile of patients with high-DSA

The profiles of the eight patients in the DSA-high group are presented in Table 3. They were mainly females (7/8), suggesting alloantibody production due to pregnancy. Although only two patients (#4 and #6) received a DSA-depleting intervention, neutrophil engraftment was observed in seven cases. One patient (#7) who had DSA against HLA-B*52:01 (MFI 5.832) and was transplanted with a sufficient number of CD34^+^ cells (6.37*10^6^/kg) without DSA-depleting intervention developed graft failure. The patient died of sepsis on day 30 without engraftment. All three patients with MFI > 10,000 (#1: MFI, 28,302, #2: 15,742 and #3: 14,441) achieved neutrophil engraftment on days 10, 14, and 10, respectively, without DSA-depleting intervention. Patient 1 died of early recurrence within 28 days. The remaining two patients (2 and 3) developed recurrence at 90 and 132 days, respectively. Although detailed clarification concerning the process of donor selection and the indications for DSA-depleting interventions was desired, further information was unavailable due to data availability restrictions.

**Table 3:**
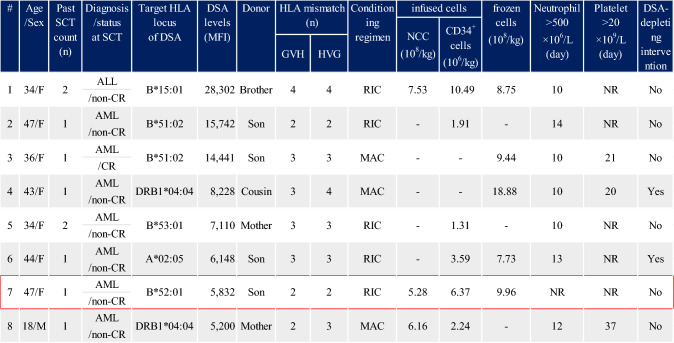
Profile and engraftment outcome of eight patients with high DSA

SCT, stem cell transplantation; HLA, human leukocyte antigen; DSA, donor-specific anti-HLA antibody; MFI, median fluorescence intensity; GVH, in graft-versus-host direction; HVG, in host-versus-graft direction; NCC, nuclear cell count; ALL, acute lymphoblastic leukemia; AML, acute myeloge1nous leukemia; CR, complete remission; MAC, myeloablative conditioning; RIC, reduced-intensity conditioning; NR, not reached

During the 5 years of TRUMP registry data, eight patients (0.20%) with MFI > 5,000 in the 3,882 patients received HLA-mismatched peripheral blood stem cell transplantation. Two patients (#4 and #6) underwent DSA-depleting depletion interventions. Only one patient (#7, red square) with DSA of MFI = 5,832 against B*52:01 and transplanted with a sufficient number of CD34 + cells (6.37*106/kg) without DSA-depleting intervention developed graft failure

“-” denotes no available data

## Discussion

One remarkable finding discovered after opening the data was that the background of the anti-HLA antibody-positive group differed from that of the negative group. The predominance of females, multiple transplantations, and HLA mismatches in the anti-HLA antibody-positive group could reasonably explain alloantibody production through the intervening variables of pregnancy and multiple transfusions. However, these differences could not explain the high cumulative incidence of neutrophil engraftment in the anti-HLA antibody-positive group compared with that in the anti-HLA antibody-negative group. Multivariate analyses revealed that CD34, HLA, mismatching in HVG direction, MMF-/ + , and MTX-/ + were significant for neutrophil engraftment, while anti-HLA antibody positivity was not among the covariates, which comprised anti-HLA antibody, age, sex, HLA-mis in GVH, HLA-mis-HVG, HCT-CI, disease status (CR or non-CR at SCT), CD34^+^, MAC/RIC, CsA/TAC, MMF-/ + , and MTX-/ + . This result was also true for the preliminary analyses with thresholds of MFI 3,000 and 1,000 (Supplemental data). This led us to assume that the better neutrophil engraftment in the anti-HLA antibody-positive group was due to confounding factors. Indeed, the presence or absence of HLA antibodies correlated with MTX use, and the difference between the effect of HLA antibodies disappeared in the MTX-adjusted cumulative incidence function (CIF). Since GVHD prophylaxis using TAC plus steroids in haploidentical transplantation has been widespread in Japan [11], prior information about the presence of HLA antibodies may have prevented a myelosuppressive MTX regimen. Regarding platelet engraftment, the presence of DSA has a slight negative effect on platelet engraftment. In multivariate analysis, this effect was observed only for DSA with MFI more than 5,000 and was not a significant factor for MFI 3,000 and 1,000 (Supplemental data). In contrast, HCT-CI and CD34^+^ were extracted as significant factors regardless of the MFI threshold. This suggests that the EBMT proposal of MFI 5,000 is reasonable as a “clinically significant” cut-off.

The impact of antibodies against loci other than HLA-A, B, C, and DRB1, specifically anti-HLA-DP, DQ, and DR3/4/5, should also be noted. In cord blood transplantation, the Toranomon Hospital group reported that HLA antibodies against HLA-DP, DQ, and DR3/4/5 adversely affect engraftment and nonrelapse mortality in their institute [12, 13]. The negative effect of HLA antibodies against HLA-DP and DQ was also documented in the cord blood transplantation provided by the Japanese Red Cross Kinki Cord Blood Bank [14]. In this study, donor HLA profiles were unavailable and were omitted from the analysis; however, future analyses including these profiles are warranted.

We found that only eight patients with high-DSA levels were registered in HLA-mismatched-related PBSCT in the TRUMP data. This Fig. (8/3,882 [0.2%]) is much smaller than expected (10–24%) based on the previously reported DSA prevalence [15]. This suggests that transplants with high DSA have been avoided, because the negative risk of DSA has become widely known. We found that seven of eight patients with high DSA (MFI > 5,000) achieved neutrophil engraftment. Conveying this fact is one of the objectives of this study. It remains unclear why some cases were engrafted even with high DSA levels. The biological hierarchy of anti-HLA antibodies in vivo is as follows: complement-binding IgG, viable cell-binding IgG, and flow bead-binding IgG. Ciurea et al. claimed that complement-dependent cytotoxicity, rather than antibody-dependent cell-mediated cytotoxicity, is a mainstay of graft rejection and that the presence of complement-binding IgG has the greatest impact on engraftment. However, methods for detecting complement-binding IgG (C1q assays) are not widely available [16, 9]. Viable cell-binding IgG, the second priority, is detected by physical cross-matching. Owing to the complexity of preparing viable donor cells, this method has recently been replaced with virtual cross-matching using flow beads. However, HLA coated on flow beads does not fully reproduce HLA expressed on viable cells, and the flow bead-based method also detects IgG that binds to cryptic epitopes not expressed on intact HLA in vivo [17]. This may explain why DSA-depleting therapy using transfusion of targeted HLA-expressing platelets may or may not decrease the flow-bead-based MFI [18]. A more fundamental problem is the MFI value. The flow bead method involves a mixture of several hundred colored single-HLA antigen-coated beads in one tube and the application of a serum sample; an anti-HLA antibody is an anti-HLA epitope (or eplet) antibody [19], for example, an antibody that binds to an HLA-A*02:01-coated bead is a mixture of antibodies to multiple eplets of A*02:01 (e.g., 9F, 44RM, 62GE, 65RA, 66 K) (http://www.epregistry.com.br). The MFI value of HLA-A*02:01 reflected the sum of these antibodies. Since most eplets are shared by multiple HLAs, antibodies in the sample will also bind to beads of other HLAs sharing eplets with A*02:01, resulting in an underestimation of the MFI value per bead (shared epitope phenomenon) [20]. Therefore, it should be recognized that the MFI value is only one indicator of the true amount of antibodies. In this series, except for B*15:01 at #1 and B*52:01 at #7 in the DSA-high group, the remaining HLA types are rare among Japanese individuals. Thus, it was considered that these antibodies might be natural antibodies. Natural antibodies, represented by flow bead-binding IgG among the aforementioned three, do not bind in vivo and therefore do not contribute to rejection.

In conclusion, contrary to previous reports, the effects of anti-HLA antibodies on engraftment were inconclusive. A limitation of the present study is that the sample population was already skewed owing to selection bias, and no valid statistical analysis was possible. However, it might be beneficial to determine the presence of patients with anecdotal cancer who achieved neutrophil engraftment despite high-DSA levels through a nationwide survey. Simultaneously, selecting patients who require DSA-depleting interventions is the next challenge.

## Supplementary Information

Below is the link to the electronic supplementary material.

Supplementaryfile1 (DOCX 25 KB)

## Data Availability

Data are available from the corresponding author upon reasonable request.

## References

[1] Takanashi M, Atsuta Y, Fujiwara K, Kodo H, Kai S, Sato H, Kohsaki M, Azuma H, Tanaka H, Ogawa A, et al. The impact of anti-HLA antibodies on unrelated cord blood transplantations. Blood. 2010;116(15):2839–46. 20628152 10.1182/blood-2009-10-249219

[2] Yoshihara S, Maruya E, Taniguchi K, Kaida K, Kato R, Inoue T, Fujioka T, Tamaki H, Ikegame K, Okada M, et al. Risk and prevention of graft failure in patients with preexisting donor-specific HLA antibodies undergoing unmanipulated haploidentical SCT. Bone Marrow Transplant. 2012;47(4):508–15. 21691261 10.1038/bmt.2011.131

[3] Chang YJ, Zhao XY, Xu LP, Zhang XH, Wang Y, Han W, Chen H, Wang FR, Mo XD, Zhang YY, et al. Donor-specific anti-human leukocyte antigen antibodies were associated with primary graft failure after unmanipulated haploidentical blood and marrow transplantation: a prospective study with randomly assigned training and validation sets. J Hematol Oncol. 2015;8:84. 26156584 10.1186/s13045-015-0182-9 PMC4496923

[4] Bramanti S, Calafiore V, Longhi E, Mariotti J, Crespiatico L, Sarina B, De Philippis C, Nocco A, Santoro A, Castagna L. Donor-Specific Anti-HLA Antibodies in Haploidentical Stem Cell Transplantation with Post-Transplantation Cyclophosphamide: Risk of Graft Failure, Poor Graft Function, and Impact on Outcomes. Biol Blood Marrow Transplant. 2019;25(7):1395–406. 30826463 10.1016/j.bbmt.2019.02.020

[5] Ciurea SO, Al Malki MM, Kongtim P, Zou J, Aung FM, Rondon G, Chen J, Taniguchi M, Otoukesh S, Nademanee A, et al. Treatment of allosensitized patients receiving allogeneic transplantation. Blood Adv. 2021;5(20):4031–43. 34474478 10.1182/bloodadvances.2021004862 PMC8945639

[6] Chang YJ, Xu LP, Wang Y, Zhang XH, Chen H, Chen YH, Wang FR, Han W, Sun YQ, Yan CH, et al. Rituximab for desensitization during HLA-mismatched stem cell transplantation in patients with a positive donor-specific anti-HLA antibody. Bone Marrow Transplant. 2020;55(7):1326–36. 32385341 10.1038/s41409-020-0928-z

[7] Yamashita T, Ikegame K, Kojima H, Tanaka H, Kaida K, Inoue T, Ogawa H. Effective desensitization of donor-specific HLA antibodies using platelet transfusion bearing targeted HLA in a case of HLA-mismatched allogeneic stem cell transplantation. Bone Marrow Transplant. 2017;52(5):794–6. 28165448 10.1038/bmt.2017.10

[8] Zhu J, Wang Q, Liu Y, Dong Y, Liang Z, Yin Y, Liu W, Xu W, Sun Y, Wang B, et al. High-Dose immunoglobulin Intervention as an effective and simple strategy for donor specific Anti-HLA antibody desensitization in haploidentical transplant. Int Immunopharmacol. 2023;120:110299. 37201405 10.1016/j.intimp.2023.110299

[9] Ciurea SO, Cao K, Fernandez-Vina M, Kongtim P, Malki MA, Fuchs E, Luznik L, Huang XJ, Ciceri F, Locatelli F, et al. The European Society for Blood and Marrow Transplantation (EBMT) Consensus Guidelines for the Detection and Treatment of Donor-specific Anti-HLA Antibodies (DSA) in Haploidentical Hematopoietic Cell Transplantation. Bone Marrow Transplant. 2018;53(5):521–34. 29335625 10.1038/s41409-017-0062-8 PMC7232774

[10] Kanda Y. Investigation of the freely available easy-to-use software “EZR” for medical statistics. Bone Marrow Transplant. 2013;48(3):452–8. 23208313 10.1038/bmt.2012.244 PMC3590441

[11] Ikegame K, Yoshida T, Yoshihara S, Daimon T, Shimizu H, Maeda Y, Ueda Y, Kaida K, Ishii S, Taniguchi K, et al. Unmanipulated Haploidentical Reduced-Intensity Stem Cell Transplantation Using Fludarabine, Busulfan, Low-Dose Antithymocyte Globulin, and Steroids for Patients in Non-Complete Remission or at High Risk of Relapse: A Prospective Multicenter Phase I/II Study in Japan. Biol Blood Marrow Transplant. 2015;21(8):1495–505. 25921715 10.1016/j.bbmt.2015.04.012

[12] Yamamoto H, Uchida N, Matsuno N, Ota H, Kageyama K, Wada S, Kaji D, Nishida A, Ishiwata K, Takagi S, et al. Anti-HLA antibodies other than against HLA-A, -B, -DRB1 adversely affect engraftment and nonrelapse mortality in HLA-mismatched single cord blood transplantation: possible implications of unrecognized donor-specific antibodies. Biol Blood Marrow Transplant. 2014;20(10):1634–40. 24972251 10.1016/j.bbmt.2014.06.024

[13] Osada M, Yamamoto H, Watanabe O, Yamaguchi K, Kageyama K, Kaji D, Taya Y, Nishida A, Ishiwata K, Takagi S, et al. Lymphocyte Crossmatch Testing or Donor HLA-DP and -DQ Allele Typing Effectiveness in Single Cord Blood Transplantation for Patients With Anti-HLA Antibodies Other Than Against HLA-A, -B, -C, and -DRB1. Transplant Cell Ther. 2024;30(7):696.e691-696.e614. 10.1016/j.jtct.2024.04.011 38641011

[14] Jo T, Arai Y, Hatanaka K, Ishii H, Ono A, Matsuyama N, Mori J, Koh Y, Azuma F, Kimura T. Adverse effect of donor-specific anti-human leukocyte antigen (HLA) antibodies directed at HLA-DP/-DQ on engraftment in cord blood transplantation. Cytotherapy. 2023;25(4):407–14. 36335019 10.1016/j.jcyt.2022.10.005

[15] Zou J, Wang T, He M, Bolon YT, Gadalla SM, Marsh SGE, Kuxhausen M, Gale RP, Sharma A, Assal A, et al. Number of HLA-Mismatched Eplets Is Not Associated with Major Outcomes in Haploidentical Transplantation with Post-Transplantation Cyclophosphamide: A Center for International Blood and Marrow Transplant Research Study. Transplant Cell Ther. 2022;28(2):107.e101-107.e108. 10.1016/j.jtct.2021.11.001 PMC8848305 34774819

[16] Ciurea SO, Thall PF, Milton DR, Barnes TH, Kongtim P, Carmazzi Y, López AA, Yap DY, Popat U, Rondon G, et al. Complement-Binding Donor-Specific Anti-HLA Antibodies and Risk of Primary Graft Failure in Hematopoietic Stem Cell Transplantation. Biol Blood Marrow Transplant. 2015;21(8):1392–8. 25985919 10.1016/j.bbmt.2015.05.001 PMC4506716

[17] El-Awar N, Terasaki PI, Nguyen A, Sasaki N, Morales-Buenrostro LE, Saji H, Maruya E, Poli F. Epitopes of human leukocyte antigen class I antibodies found in sera of normal healthy males and cord blood. Hum Immunol. 2009;70(10):844–53. 19580837 10.1016/j.humimm.2009.06.020

[18] Zhang R, He Y, Yang D, Jiang E, Ma Q, Pang A, Zhai W, Wei J, Feng S, Han M. Combination treatment of rituximab and donor platelets infusion to reduce donor-specific anti-HLA antibodies for stem cells engraftment in haploidentical transplantation. J Clin Lab Anal. 2020;34(7): e23261. 32112480 10.1002/jcla.23261 PMC7370703

[19] Nishikawa K, Masui S, Ishida H. Virtual crossmatching and epitope analysis in kidney transplantation: What the physician involved in kidney transplantation should know? Int Urol. 2023;30(1):7–19. 10.1111/iju.15059 36194790

[20] Garcia-Sanchez C, Usenko CY, Herrera ND, Tambur AR. The shared epitope phenomenon-A potential impediment to virtual crossmatch accuracy. Clin Transplant. 2020;34(8): e13906. 32418254 10.1111/ctr.13906

