# Supplementary material for: Engraftment outcome of patients with anti-HLA antibodies in HLA-mismatched peripheral blood stem cell transplantation
Source: Int J Hematol. 2025 Mar 20;121(6):848–56. doi: 10.1007/s12185-025-03952-y (PMC12106477; doi:10.1007/s12185-025-03952-y)
Supplement: Supplementary file 1 — Supplementaryfile1 (DOCX 25 KB) [file 12185_2025_3952_MOESM1_ESM.docx]

|  | **Definition of High-DSA group (n=8) with MFI 5,000 as threshold** | | | | | | |
| --- | --- | --- | --- | --- | --- | --- | --- |
|  | **Neutrophil engraftment** | | |  | **Platelet engraftment** | | |
| variables | HR | 95%CI | p-value |  | HR | 95%CI | p-value |
| HLA antibody group | 0.974 | 0.772-1.228 | 0.830 |  | 0.752 | 0.566-0.998 | 0.049 |
| Age | 1.002 | 0.997-1.007 | 0.360 |  | 1.005 | 0.999-1.010 | 0.068 |
| Sex | 0.877 | 0.761-1.035 | 0.130 |  | 0.914 | 0.766-1.091 | 0.320 |
| HLA mismatch (GVH) | 0.964 | 0.899-1.047 | 0.390 |  | 0.959 | 0.865-1.063 | 0.430 |
| HLA mismatch (HVG) | 1.094 | 1.008-1.188 | 0.032 |  | 0.936 | 0.851-1.031 | 0.180 |
| HCT-CI | 0.966 | 0.924-1.011 | 0.140 |  | 0.910 | 0.862-0.961 | <0.001 |
| CR/nonCR | 0.956 | 0.838-1.091 | 0.500 |  | 0.981 | 0.836-1.151 | 0.820 |
| CD34^+^ | 1.031 | 1.003-1.059 | 0.031 |  | 1.040 | 1.009-1.072 | 0.011 |
| MAC/RIC | 0.909 | 0.776-1.065 | 0.240 |  | 1.060 | 0.871-1.290 | 0.560 |
| Cyclosporine | 0.773 | 0.476-1.254 | 0.300 |  | 0.772 | 0.453-1.315 | 0.340 |
| Tacrolimus | 0.881 | 0.552-1.408 | 0.600 |  | 0.874 | 0.530-1.440 | 0.600 |
| Mycophenolate mofetil | 0.593 | 0.471-0.748 | <0.001 |  | 0.974 | 0.741-1.282 | 0.850 |
| methotrexate | 0.439 | 0.368-0.523 | <0.001 |  | 1.108 | 0.900-1.364 | 0.330 |
|  |  |  |  |  |  |  |  |
|  | **Definition of High-DSA group (n=14) with MFI 3,000 as threshold** | | | | | | |
|  | **Neutrophil engraftment** | | |  | **Platelet engraftment** | | |
| variables | HR | 95%CI | p-value |  | HR | 95%CI | p-value |
| HLA antibody group | 0.991 | 0.713-1.378 | 0.830 |  | 1.143 | 0.778-1.677 | 0.500 |
| Age | 1.002 | 0.997-1.007 | 0.360 |  | 1.004 | 0.999-1.010 | 0.100 |
| Sex | 0.889 | 0.764-1.035 | 0.130 |  | 0.939 | 0.786-1.123 | 0.490 |
| HLA mismatch (GVH) | 0.965 | 0.889-1.047 | 0.390 |  | 0.959 | 0.865-1.063 | 0.430 |
| HLA mismatch (HVG) | 1.094 | 1.007-1.187 | 0.032 |  | 0.934 | 0.849-1.029 | 0.170 |
| HCT-CI | 0.966 | 0.924-1.010 | 0.140 |  | 0.904 | 0.857-0.955 | <0.001 |
| CR/nonCR | 0.957 | 0.839-1.092 | 0.500 |  | 0.987 | 0.841-1.158 | 0.880 |
| CD34^+^ | 1.031 | 1.003-1.059 | 0.031 |  | 1.043 | 1.011-1.075 | 0.007 |
| MAC/RIC | 0.910 | 0.776-1.068 | 0.240 |  | 1.063 | 0.874-1.293 | 0.540 |
| Cyclosporine | 0.773 | 0.476-1.255 | 0.300 |  | 0.777 | 0.454-1.331 | 0.360 |
| Tacrolimus | 0.880 | 0.551-1.404 | 0.600 |  | 0.865 | 0.522-1.434 | 0.570 |
| Mycophenolate mofetil | 0.594 | 0.472-0.748 | <0.001 |  | 0.989 | 0.753-1.298 | 0.940 |
| methotrexate | 0.440 | 0.370-0.524 | <0.001 |  | 1.141 | 0.928-1.401 | 0.210 |
|  |  |  |  |  |  |  |  |
|  | **Definition of High-DSA group (n=30) with MFI 1,000 as threshold** | | | | | | |
|  | **Neutrophil engraftment** | | |  | **Platelet engraftment** | | |
| variables | HR | 95%CI | p-value |  | HR | 95%CI | p-value |
| HLA antibody group | 1.002 | 0.767-1.307 | 0.990 |  | 1.127 | 0.834-1.521 | 0.440 |
| Age | 1.002 | 0.997-1.007 | 0.370 |  | 1.004 | 0.999-1.010 | 0.100 |
| Sex | 0.889 | 0.764-1.036 | 0.130 |  | 0.939 | 0.786-1.123 | 0.490 |
| HLA mismatch (GVH) | 0.964 | 0.899-1.047 | 0.390 |  | 0.959 | 0.865-1.063 | 0.430 |
| HLA mismatch (HVG) | 1.094 | 1.008-1.188 | 0.032 |  | 0.935 | 0.849-1.030 | 0.170 |
| HCT-CI | 0.966 | 0.924-1.010 | 0.130 |  | 0.904 | 0.857-0.954 | <0.001 |
| CR/nonCR | 0.957 | 0.839-1.092 | 0.520 |  | 0.987 | 0.841-1.158 | 0.880 |
| CD34^+^ | 1.031 | 1.003-1.059 | 0.028 |  | 1.043 | 1.011-1.075 | 0.007 |
| MAC/RIC | 0.910 | 0.776-1.068 | 0.250 |  | 1.064 | 0.875-1.293 | 0.530 |
| Cyclosporine | 0.773 | 0.477-1.254 | 0.300 |  | 0.777 | 0.454-1.332 | 0.360 |
| Tacrolimus | 0.879 | 0.551-1.404 | 0.590 |  | 0.865 | 0.522-1.435 | 0.580 |
| Mycophenolate mofetil | 0.594 | 0.472-0.748 | <0.001 |  | 0.988 | 0.752-1.298 | 0.930 |
| methotrexate | 0.440 | 0.370-0.524 | <0.001 |  | 1.140 | 0.928-1.400 | 0.210 |
|  |  |  |  |  |  |  |  |
| Supplemental data. Multivariate (Fine and Gray) analyses for neutrophil and platelet engraftments. High-DSA group was defined with MFI 5,000, 3,000, and 1,000 as threshold. HLA mismatch (GVH): number of HLA mismatches in GVH direction; HLA mismatch (HVG): number of HLA mismatches in HVG direction; HCT-CI: Hematopoietric Cell Transplantation-specific Comorbidity Index; MAC/RIC: myeloablative conditioning or reduced intensity conditioning | | | | | | | |
